# Immune persistence after different polio sequential immunization schedules in Chinese infants

**DOI:** 10.1038/s41541-024-00831-1

**Published:** 2024-02-29

**Authors:** Ting Zhao, Jing Li, Teng Huang, Zhi-Fang Ying, Yan-Chun Che, Zhi-Mei Zhao, Yu-Ting Fu, Jun-Hui Tao, Qing-Hai Yang, Ding-Kai Wei, Guo-Liang Li, Li Yi, Yu-Ping Zhao, Hong-Bo Chen, Jian-Feng Wang, Rui-Ju Jiang, Lei Yu, Wei Cai, Wei Yang, Ming-Xue Xie, Qiong-Zhou Yin, Jing Pu, Li Shi, Chao Hong, Yan Deng, Lu-Kui Cai, Jian Zhou, Yu Wen, Hong-Sen Li, Wei Huang, Zhao-Jun Mo, Chang-Gui Li, Qi-Han Li, Jing-Si Yang

**Affiliations:** 1 Institute of Medical Biology, Chinese Academy of Medical Sciences and Peking Union Medical College, Kunming, China; 2 Guangxi Province Center for Disease Control and Prevention, Nanning, China; 3 National Institutes for Food and Drug Control, Beijing, China; 4 Liujiang District Center for Disease Prevention and Control, Liuzhou, China; 5 Liucheng County Center for Disease Prevention and Control, Liuzhou, China; 6 Rongan County Center for Disease Prevention and Control, Liuzhou, China

**Keywords:** Public health, Clinical trial design

## Abstract

Trivalent oral poliovirus vaccine (tOPV) has been withdrawn and instead an inactivated poliovirus vaccine (IPV) and bivalent type 1 and type 3 OPV (bOPV) sequential immunization schedule has been implemented since 2016, but no immune persistence data are available for this polio vaccination strategy. This study aimed to assess immune persistence following different polio sequential immunization schedules. Venous blood was collected at 24, 36, and 48 months of age from participants who had completed sequential schedules of combined IPV and OPV in phase III clinical trials. The serum neutralizing antibody titers against poliovirus were determined, and the poliovirus-specific antibody-positive rates were evaluated. A total of 1104 participants were enrolled in this study. The positive rates of poliovirus type 1- and type 3-specific antibodies among the sequential immunization groups showed no significant difference at 24, 36, or 48 months of age. The positive rates of poliovirus type 2-specific antibody in the IPV-IPV-tOPV group at all time points were nearly 100%, which was significantly higher than the corresponding rates in other immunization groups (IPV-bOPV-bOPV and IPV-IPV-bOPV). Immunization schedules involving one or two doses of IPV followed by bOPV failed to maintain a high positive rate for poliovirus type 2-specific antibody.

## Introduction

As we approach the end of poliomyelitis (polio) eradication, cases caused by wild-type polio are decreasing, whereas vaccine-derived poliovirus (VDPV) cases caused by oral poliovirus vaccine (OPV) are predominant^1^ To reduce the cases of VDPV, it is imperative that OPV is phased out, and countries fully convert to an inactivated poliovirus vaccine (IPV) immunization^2^. Starting from May 1, 2016, developing countries, including China, implemented a new polio vaccine immunization strategy, discontinuing trivalent live attenuated polio vaccine (tOPV) and updating the polio immunization regimen to include one dose of inactivated polio vaccine (IPV) plus two doses of bivalent live attenuated polio vaccine (bOPV)^2–4^.

The persistence of poliovirus-specific antibody levels in individuals who receive combined sequential immunization with IPV and bOPV after switching immunization strategies is relevant to the prevention and control of wild-type polio cases and VDPV. Prior to the switching of immunization strategies, some studies focused on the immune persistence of combined sequential immunization with IPV and trivalent OPV^5^ and the immune persistence of combined vaccines containing IPV components^6,7^. To date, many clinical trials have investigated the safety and immunogenicity of IPV and bOPV combined sequential immunization^8–10^, but few studies have investigated its immune persistence. At present, the immunization policy of most developing countries, including China, is to use combined sequential immunization with IPV and bOPV for fundamental immunity and bOPV for booster immunization^3,11^. Whether such an immunization strategy and the timing of booster immunization are appropriate requires further investigation.

We completed a phase III clinical trial before tOPV was discontinued in 2015–2016 to evaluate the immunogenicity and safety of bOPV and Sabin strain-based IPV (sIPV) combined sequential immunization in 2-month-old infants^9^. To further observe the long-term immunogenicity of different sequential immunization schedules combining IPV and bOPV, this follow-up extension study was conducted on participants of the phase III clinical trial. Venous blood was collected at the ages of 24, 36, and 48 months from participants who had completed basic polio immunization, the serum was separated to determine the titers of neutralizing antibodies against poliovirus types 1, 2, and 3, and the antibody-positive rates and antibody levels were evaluated.

Through this clinical investigation, we were able to observe sustained levels of polio-neutralizing antibodies, particularly type 2 polio-neutralizing antibodies, in individuals who received a sequential immunization program with one dose of IPV and two doses of IPV. At present, China and some developing countries use sIPV for immunization^12,13^, and the difference in immune persistence between sIPV and Salk strain-based IPV (known as conventional IPV, cIPV) can also be reflected in this study. The investigation of immune persistence after fundamental immunization in infants and young children can help us find the appropriate timing of booster immunization and explain whether a single dose of bOPV at 48 months of age is justified in the current polio immunization strategy.

## Results

### Study population

**Fig. 1:**
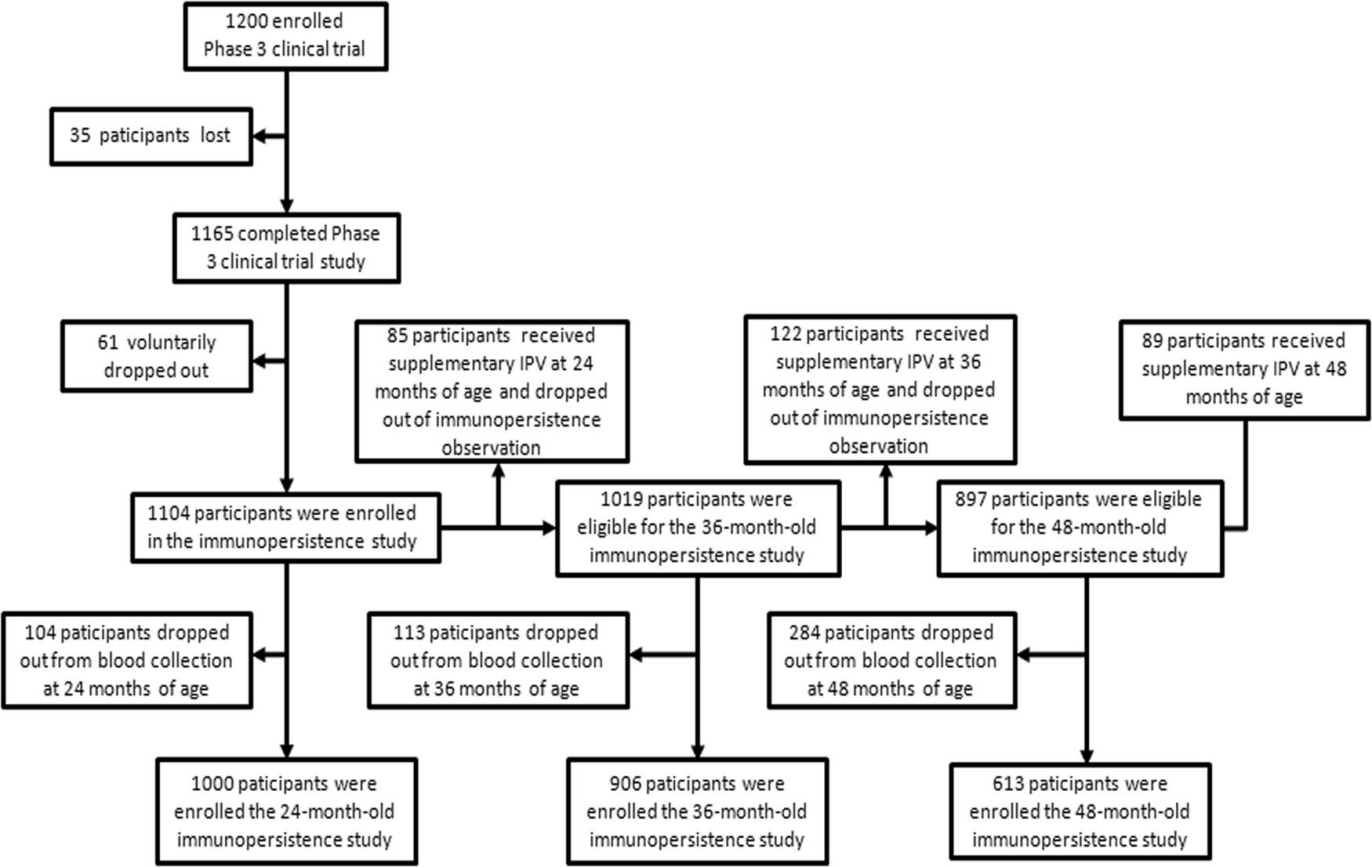
Profile of the immune persistence study. IPV inactivated polio vaccine.

Of the 1200 participants in the previous phase III clinical trial, 35 did not receive all three doses of polio vaccine or lacked valid serum neutralizing antibody titer data; another 61 individuals voluntarily withdrew from the clinical study owing to a variety of reasons, such as moving away. The remaining 1104 participants enrolled in the present clinical study on the immunopersistence of sequential schedules of combined IPV and bOPV. In the previous trial, these 1104 participants had been assigned to six different immunization schedules: sIPV-bOPV-bOPV, sIPV-sIPV-bOPV, sIPV-sIPV-tOPV, cIPV-bOPV-bOPV, cIPV-cIPV-bOPV, or cIPV-cIPV-tOPV. The participant characteristics were compared among polio immunization schedule groups; there were no significant differences in participant age, sex, or race composition among groups (Table 1). At 24 months of age, 104 of the 1104 participants withdrew from blood collection; the other 1000 participants completed the 24-month-old immunopersistence observation. To compensate for the deficiency of poliovirus type 2-specific antibody in some participants, a single dose of IPV was administered to participants whose previous serum test revealed a poliovirus type 2-specific neutralizing antibody titer of <8. In total, a single dose of IPV was administered at 24 months of age to 85 participants who had a poliovirus type 2-specific antibody titer of <8 after primary immunization. Importantly, these individuals who were revaccinated with IPV were no longer included in the subsequent immunopersistence study. Thus, 1019 of the original 1104 participants were eligible for the 36-month-old immunopersistence observation. Of these 1019 participants, 113 participants withdrew; 906 participants completed the blood collection and antibody titer detection at 36 months of age. On the basis of those results, 122 participants received an additional dose of IPV after their 36-month blood collection, leaving 897 participants enrolled in the 48-month immunopersistence study. Finally, 284 participants withdrew over this final period; 613 participants completed the blood collection and antibody titer detection at 48 months of age (Fig. 1). The number of participants who received a supplementary dose of IPV in each group at the ages of 24, 36, and 48 months is shown in Supplementary Table 1-1, and the number of participants who completed blood collection for the observation of immune persistence is shown in Supplementary Table 1-2.

**Table 1:** Characteristics of participants subjected to different polio immunization schedules

	sIPV-bOPV-bOPV	cIPV-bOPV-bOPV	cIPV-cIPV-bOPV	sIPV-sIPV-tOPV	sIPV-sIPV-bOPV	cIPV-cIPV-tOPV	*p* value
	(*n* = 188)	(*n* = 183)	(*n* = 183)	(*n* = 180)	(*n* = 186)	(*n* = 184)	
Age (years), mean ± SD	2.6 ± 0.1	2.6 ± 0.1	2.6 ± 0.1	2.6 ± 0.1	2.6 ± 0.1	2.6 ± 0.1	0.99
Sex, *n* (%)							
Male	107 (56.9)	90 (49.2)	105 (57.4)	89 (49.4)	100 (53.8)	97 (52.7)	0.46
Female	81 (43.1)	93 (50.8)	78 (42.6)	91 (50.6)	86 (46.2)	87 (47.3)	
Race, *n* (%)							
Han	62 (33%)	53 (33%)	59 (32.2%)	48 (26.7%)	50 (26.9%)	62 (33.7%)	0.5
Zhuang	113 (60.1%)	115 (62.8%)	111 (60.7%)	116 (64.4%)	121 (65.1%)	106 (57.6%)	0.7
Miao	4 (2.1%)	4 (2.2%)	4 (2.2%)	3 (1.7%)	4 (2.2%)	5 (2.7%)	1
Yao	1 (0.5%)	1 (0.5%)	3 (1.6%)	2 (1.1%)	1 (0.5%)	0 (0%)	0.5
Other	8 (4.3%)	10 (5.5%)	6 (3.3%)	11 (6.1%)	10 (5.4%)	11 (6%)	0.8

*tOPV* trivalent oral polio vaccine, *cIPV* conventional inactivated poliovirus vaccine, *bOPV* bivalent oral polio vaccine, *sIPV* Sabin strain–based inactivated poliovirus vaccine, *SD* standard deviation.

### Comparison of poliovirus-specific antibody levels between different sequential immunization programs at the same time point

The serum poliovirus-specific neutralizing antibody titers were measured at 24, 36, and 48 months of age. A statistical analysis was conducted to compare the levels of poliovirus-specific neutralizing antibodies at 24, 36, or 48 months among participants who received different sequential polio immunizations. The positive rates for poliovirus type 1-specific antibody did not differ significantly among the sequential immunization groups (Table 2). The positive rates for poliovirus type 3-specific antibody did not differ significantly among the groups at 24 or 48 months of age, but a slight difference was observed at 36 months of age (Table 2, Supplemental Table 2).

**Table 2:** Seropositive rate and geometric mean titer against poliovirus types 1, 2, and 3 at 24, 36, and 48 months of age following different immunization schedules

	sIPV-bOPV-bOPV	cIPV-bOPV-bOPV	cIPV-cIPV-bOPV	sIPV-sIPV-tOPV	sIPV-sIPV-bOPV	cIPV-cIPV-tOPV	*P* value	Test method
24 months								
Type 1								
Seropositive, *n* (%)	173 (100%)	170 (100%)	158 (99.40%)	162 (99.40%)	169 (100%)	166 (100%)	0.218	Fisher exact
GMT (95% CI)	938.4 (794.6–1108)	604.5 (515.5–708.7)	453 (372.9–550.2)	473.8 (386.7–580.6)	814.4 (679.2–976.4)	280.5 (232.1–339.1)	<0.0001	ANOVA
Type 2								
Seropositive, *n* (%)	62 (35.80%)	125 (73.50%)	143 (89.9%)	163 (100%)	143 (84.60%)	165 (99.4%)	<0.0001	Pearson Chi-Square
GMT (95% CI)	7.1 (6.15–8.2)	18.27 (15–22.26)	33.7 (27.8–40.8)	284.2 (241.4–334.5)	28.66 (23.59–34.82)	266.6 (225.5–315.2)	<0.0001	Kruskal–Wallis test
Type 3								
Seropositive, *n* (%)	173 (100%)	170 (100%)	157 (98.7%)	160 (98.20%)	166 (98.20%)	162 (97.6%)	0.111	Fisher exact
GMT (95% CI)	216.5 (181.9–257.6)	214.9 (183.2–252)	279.3 (230.1–339)	189.9 (151–238.7)	285.5 (237–343.8)	185.5 (147.7–232.9)	0.0017	Kruskal–Wallis test
36 months								
Type 1								
Seropositive, *n* (%)	121 (100%)	135 (100%)	161 (99.40%)	164 (99.40%)	160 (100%)	161 (98.8%)	0.712	Fisher exact
GMT (95% CI)	821.8 (681.1–991.6)	500 (416.3–600.4)	425 (351.2–514.3)	446.9 (361.6–552.4)	798.3 (670.1–951.1)	227.2 (184.4–279.8)	<0.0001	Kruskal–Wallis test
Type 2								
Seropositive, *n* (%)	47 (38.8%)	104 (77.0%)	148 (91.4%)	164 (99.4%)	137 (85.6%)	162 (99.4%)	<0.0001	Pearson Chi-Square
GMT (95% CI)	8.1 (6.6–10)	22.14 (17.6–27.9)	38.8 (32–47.1)	312.6 (263.4–371)	31.8 (25.8–39.1)	287.9 (242.3–342.2)	<0.0001	Kruskal–Wallis test
Type 3								
Seropositive, *n* (%)	121 (100%)	135 (100%)	162 (100%)	159 (96.4%)	155 (96.9%)	157 (96.3%)	0.003	Fisher exact
GMT (95% CI)	168.4 (134.9–210.3)	194.6 (162.7–232.8)	256.2 (215.2–305)	162 (130.4–201.4)	254.5 (208–311.5)	151.9 (121–190.6)	<0.0001	Kruskal–Wallis test
48 months								
Type 1								
Seropositive, *n* (%)	63 (100%)	95 (100%)	111 (99.1%)	118 (100%)	105 (100%)	119 (99.2%)	0.925	Fisher exact
GMT (95% CI)	1214 (948.6–1554)	561.9 (452.7–697.3)	420 (332.6–530.3)	511.8 (409.7–639.3)	799.9 (629.5–1016)	258.1 (204.6–325.7)	<0.0001	ANOVA
Type 2								
Seropositive, *n* (%)	37 (58.7%)	78 (82.1%)	99 (88.4%)	118 (100%)	85 (81%)	119 (99.2%)	<0.0001	Pearson Chi-Square
GMT (95% CI)	12 (8.8–16.4)	21.48 (16.56–27.88)	28.5 (22.9–35.5)	186.5 (156.9–221.8)	23.23 (18.58–29.04)	184.9 (153–223.5)	<0.0001	Kruskal–Wallis test
Type 3								
Seropositive, *n* (%)	63 (100%)	95 (100%)	112 (100%)	117 (99.2%)	104 (99%)	120 (100%)	0.715	Fisher exact
GMT (95% CI)	249.7 (180–346.2)	208.6 (166.5–261.2)	275.3 (224.1–338.2)	184.1 (146.2–231.7)	294.7 (243–357.3)	165.4 (131.2–208.4)	0.0006	Kruskal–Wallis test

If there are significant differences among groups, the pairwise comparisons are shown in the Supplementary Tables 2 and 3.

*tOPV* trivalent oral polio vaccine, *cIPV* conventional inactivated poliovirus vaccine, *bOPV* bivalent oral polio vaccine, *sIPV* Sabin strain–based inactivated poliovirus vaccine, *CI* confidence interval, *GMT* geometric mean titer.

By contrast, the positive rates for poliovirus type 2-specific antibody in serum collected at 24, 36, and 48 months of age differed significantly among the sequential immunization groups. The positive rates for poliovirus type 2-specific antibody were nearly 100% in the sIPV-sIPV-tOPV and cIPV-cIPV-tOPV groups at all time points; these positive rates were significantly higher than those in the other immunization groups. The positive rates for poliovirus type 2-specific antibody were significantly lower in the groups with an immunization schedule that contained only a single dose of IPV (sIPV-bOPV-bOPV, cIPV-bOPV-bOPV) than in those with an immunization schedule that contained two doses of IPV (cIPV-cIPV-bOPV, sIPV-sIPV-bOPV) (Table 2, Supplementary Table 2). In addition, for the immunization schedules with only one dose of IPV, the positive rates of poliovirus type 2-specific antibody in the group that injected cIPV were higher than those in the group that rejected sIPV at 24, 36, and 48 months of age separately (cIPV-bOPV-bOPV > sIPV-bOPV-bOPV, Table 2, Supplementary Table 2).

The GMTs of poliovirus-specific neutralizing antibody differed significantly among different immunization schedules (Table 2). For poliovirus type 1-specific neutralizing antibody levels: (1) at the same time point, the level of poliovirus type 1-specific neutralizing antibody was higher in the bOPV-immunized group than in the tOPV-immunized group (relative poliovirus type 1-specific neutralizing antibody levels: sIPV-bOPV-bOPV and sIPV-sIPV-bOPV > sIPV-sIPV-tOPV; cIPV-bOPV-bOPV and cIPV-cIPV-bOPV > cIPV-cIPV-tOPV); (2) within the same sequential immunization schedule, the poliovirus type 1-specific neutralizing antibody levels were higher at the same time point in the sIPV-immunized group than in the cIPV-immunized group (relative poliovirus type 1-specific neutralizing antibody levels: sIPV-bOPV-bOPV > cIPV-bOPV-bOPV; sIPV-sIPV-bOPV > cIPV-cIPV-bOPV; sIPV-sIPV-tOPV > cIPV-cIPV-tOPV) (Table 2, Supplementary Tables 3-1, 3-4, 3-7), It may be that the Sabin strain is used as a challenge strain for testing serum neutralizing antibody, causing the titer of poliovirus-specific antibody to be higher for sIPV than cIPV under the same immunization program. For poliovirus type 2-specific neutralizing antibody levels: in the case of inoculation with the same IPV (all sIPV or all cIPV), the levels were higher in the tOPV-immunized group than in the bOPV-immunized group at the same time point (relative poliovirus type 2-specific neutralizing antibody levels: sIPV-sIPV-tOPV > sIPV-sIPV-bOPV > sIPV-bOPV-bOPV; cIPV-cIPV-tOPV > cIPV-cIPV-bOPV > cIPV-bOPV-bOPV) (Table 2, Supplementary Tables 3-2, 3-5, 3-8). There was little difference in poliovirus type 3-specific neutralizing antibody levels among the groups, but the level of poliovirus type 3-specific neutralizing antibody was slightly lower in the tOPV-immunized group than in the bOPV-immunized group (sIPV-sIPV-tOPV < sIPV-sIPV-bOPV; cIPV-cIPV-tOPV < cIPV-cIPV-bOPV) (Table 2, Supplementary Tables 3-3, 3-6, 3-9).

### Changes in poliovirus-specific antibody levels along the sampling time series axis

The changes in poliovirus-specific neutralizing antibody titers were observed longitudinally, by comparing the levels at 5 (28 days after the completion of basic immunization with three doses of polio vaccine), 24, 36, and 48 months of age. In order to more effectively analyze the overall changes in antibody level over time, these analyses were conducted on the set of data from the 496 participants who had antibody titers determined for blood samples collected at 24, 36, and 48 months of age and had not been additionally vaccinated with a supplementary dose of IPV. Participants who had received booster immunization or did not have an effective serum antibody titer at 24–48 months of age were excluded. The seropositive rates and GMTs in this set of data from 496 participants are shown in Supplementary Table 4, and the results of differential analysis among the sequential immunization groups at the same time point (24, 36, and 48 months of age separately) were similar to those of the full data set above.

**Fig. 2:**
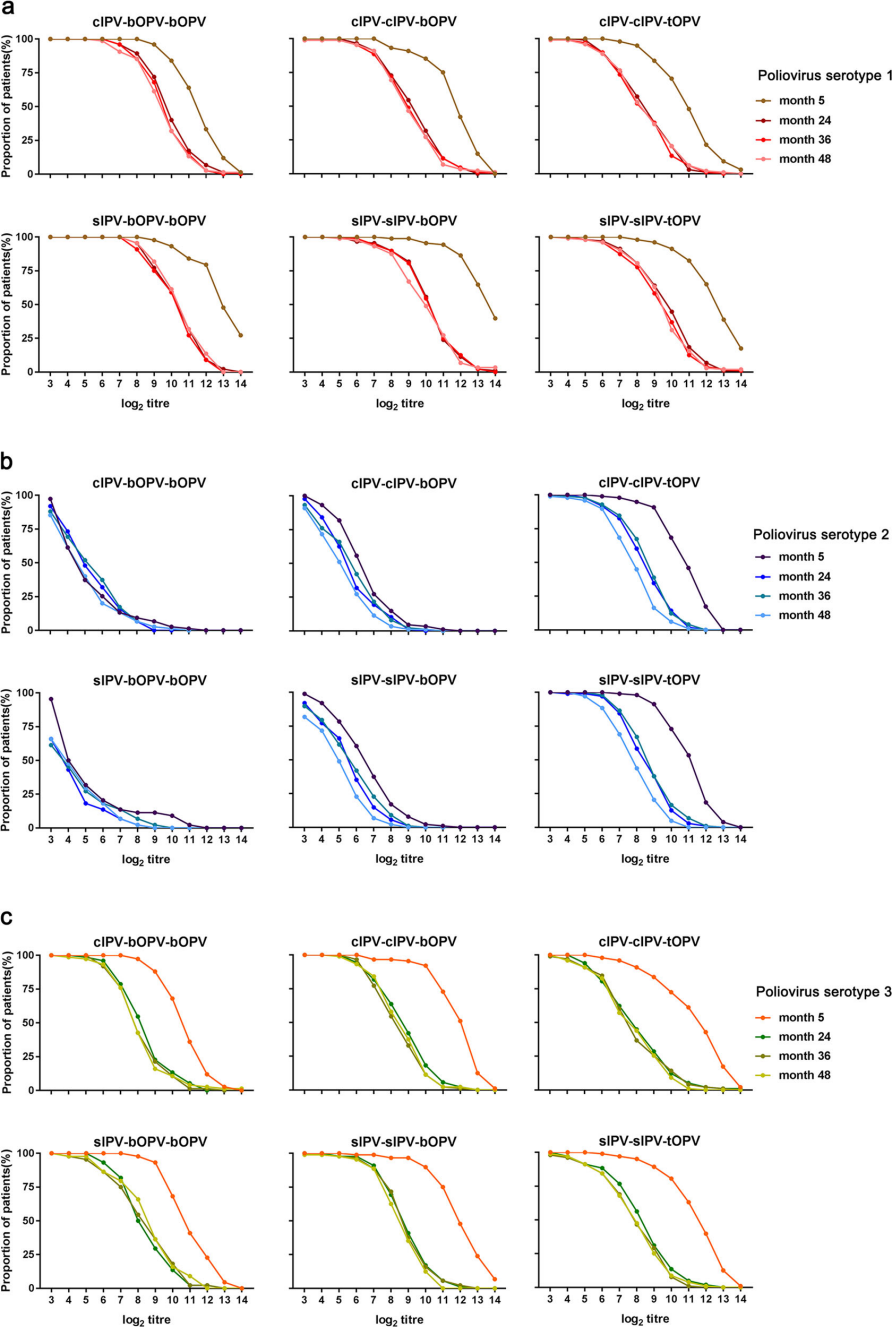
Changes in poliovirus-specific antibody levels along the sampling time series axis. Reverse cumulative distribution curves showing the distribution of serum neutralizing antibody titres against type 1 (**a**), type 2 (**b**), and type 3 (**c**) polioviruses in participants vaccinated with different immunization schedules at 5, 24, 36 and 48 months of age.

The changes in poliovirus-specific antibody titers over time in each immunization schedule group are displayed in Fig. 2. For poliovirus type 1- and type 3-specific neutralizing antibodies, the patterns of change in poliovirus-specific neutralizing antibody titers over time were similar among the different sequential immunization schedule groups (Fig. 2a, c). At 28 days after the completion of three polio vaccine doses (5 months), almost all participants had a high titer of poliovirus type 1- and type 3-specific neutralizing antibody. During the period from 5 to 24 months, the reverse distribution curves of poliovirus type 1- and type 3-specific neutralizing antibody changed significantly, with a significant decrease in the population with a high titer of poliovirus-specific neutralizing antibody. During the period from 24 to 48 months, there was no significant change in the reverse distribution curve of poliovirus type 1- and type 3-specific neutralizing antibody. During the period from 5 to 48 months, the positive rates of poliovirus type 1- and type 3-specific neutralizing antibody (>23) did not change significantly; all immunization schedule groups maintained a positive rate of almost 100%.

The patterns of change in poliovirus type 2-specific neutralizing antibody titer over time were different among the sequential immunization schedule groups (Fig. 2b). Owing to the absence of poliovirus type 2 components in bOPV, the level of poliovirus type 2-specific neutralizing antibody was low in the sIPV-bOPV-bOPV and cIPV-bOPV-bOPV groups; correspondingly, the inverse antibody distribution curve decreased rapidly. The inverse antibody distribution curves of the groups that received two doses of IPV (sIPV-sIPV-bOPV and cIPV-cIPV-bOPV) declined more slowly than those of the groups that received only one dose of IPV (sIPV-bOPV-bOPV and cIPV-bOPV-bOPV); however, the distribution curve of poliovirus type 2-specific neutralizing antibody did not plateau, and there were no significant changes in the titers during the 5–48-month period. In the groups that received two doses of IPV and one dose of tOPV (sIPV-sIPV-tOPV and cIPV-cIPV-tOPV), the reverse distribution curves of poliovirus type 2-specific neutralizing antibody tended to be high and flat, and then decrease when the titer reached a relatively large value; in addition, the curves revealed a significant difference between the 5-month time point and the other time points, with a significant decrease in the proportion of participants with a high poliovirus type 2-specific neutralizing antibody titer, while there was no significant change during the 24–48-month period.

## Discussion

Many studies^14,15^ have shown that seroconversion rates against polioviruses types 1 and 3 were non-inferior in sequential schedules containing IPV and bOPV, compared with all-IPV or all-tOPV schedule, and the levels of poliovirus type 2-specific neutralizing antibody exhibited a significant difference among different immunization schedules (IPV-bOPV-bOPV < IPV-IPV-bOPV < IPV-IPV-tOPV or tOPV-tOPV-tOPV), the results were in concordance with the observation of immune persistence. In our clinical trial, the immune persistence of poliovirus types 1 and 3-specific neutralizing antibody performed well in both of the sequential vaccination schedules. For the type 2 polio-specific neutralizing antibody positive rate observed in the persistence study, the type 2 polio-specific neutralizing antibody positive rate decreased more strongly between 24 and 48 months of age as the number of doses of type 2 polio-related vaccine received decreased. The seroprevalence of type 2 polio-specific neutralizing antibody decreased to 35.8% and 73.5% at 24 months of age in the sIPV-bOPV-bOPV and cIPV-bOPV-bOPV groups who received only one dose of type 2 polio-related vaccine. In the sIPV-sIPV-bOPV and cIPV-cIV-bOPV groups who received two doses of type 2 polio-related vaccine, the antibody positive rates decreased to 84.6% and 89.9% at 24 months of age. In the sIPV-sIPV-tOPV and cIPV-cIPV-tOPV groups vaccinated with three doses of type 2 polio-related vaccine, antibody positive rates were 100% and 99.4% at 24 months of age, respectively, and remained high at 24–48 months of age. In this clinical trial, a booster dose of IPV was administered to individuals who tested negative for polio neutralizing antibodies at 24, 36, and 48 months of age. The number of participants receiving a booster IPV dose in each group, as shown in Supplement Table 1, was in the order of IPV–bOPV–bOPV > IPV–IPV–bOPV > IPV–IPV–tOPV, which also reflected the immune persistence of each immunization schedule.

The clinical trial data suggest that vaccination with one dose of IPV does not provide long-term persistent immunity specific to type 2 poliovirus, and it is recommended that infants who have previously received one dose of IPV plus two doses of bOPV need to be vaccinated early with a vaccine containing type 2 poliovirus before 48 months of age. The clinical trial data also suggest that vaccination with two doses of IPV is more effective than a single dose of IPV for long-term persistence. Three doses of vaccine containing the type 2 polio component is optimal and will be progressively achieved as IPV production increases to address the lack of persistence of type 2 polio antibody in the population.

The clinical trial data revealed that the positive rate of type 2 polio antibody in the group vaccinated with one dose of cIPV (cIPV-bOPV-bOPV) was higher than that in the group vaccinated with one dose of sIPV (sIPV -bOPV-bOPV) during the period of 24–48 months, which may be caused by the difference in immunogenicity between the Salk and Sabin strains^16,17^. When two doses of IPV were administered, the difference in positive rates due to different types of IPV was eliminated.

After the switch of immunization strategy in 2016, the number of circulating vaccine-derived poliovirus type 2 (cVDPV2) cases has increased, triggering a public health emergency of international concern^18–21^. There are several reasons for this, including factors such as insufficient IPV production capacity, backward economic development, imperfect medical systems, religious beliefs, an IPV shortage, and low immunization coverage in some parts of the world^22,23^. Sequential immunization schedules, including IPV, cannot be established in many countries after the cessation of tOPV, resulting in a decline in population immunity against poliovirus^24,25^. Clinical studies have shown that a sequential immunization schedule of one dose of IPV plus two doses of bOPV is not sufficient to induce high level and long-lasting immune persistence of polio type 2-specific antibody.

In response to the high cost and shortage of IPV, some developing countries administer a fractional dose of IPV (fIPV)^26,27^. Studies have shown that two doses of fIPV induce the same antibody positive rate as two doses of full-dose IPV 1 month after vaccination, and that the slope of neutralizing antibody decay appears to be similar between the study groups^28^. Therefore, vaccination with fIPV is an effective method in the absence of IPV.

Despite the ongoing decline in the number of cVDPV2, the risk of international spread of cVDPV2 remains high^29^. The long distance international spread of VDPV2 between Jerusalem, London, New York, and Montreal has revealed a new risk phenomenon, namely, the evolution of vaccine-derived polioviruses in under-immunized pockets of the population who lack intestinal mucosal immunity in IPV-using countries^29–31^. Andino et al. constructed a novel live attenuated polio vaccine (nOPV), which not only succeeded OPV in rapidly stimulating the intestinal mucosal immunity of the population against polio, but also improved the genetic stability of the attenuated Sabin strain and reduced the possibility of recovering neurotoxicity, thus minimizing the risk of producing cVDPVs^32,33^. nOPV was authorized for outbreak response use under a World Health Organization Emergency Use Listing^34^. However, nOPV2 surveillance should continue for the duration of the Emergency Use Listing^35^.

The global effort to eradicate polio has made significant progress over the years, but continued vigilance and investment in immunization programs are needed to achieve the ultimate goal of a polio-free world.

## Methods

### Phase III clinical trial

This open-label, descriptive, single-centered, uncontrolled, extension study was conducted between 2015 and 2016 on selected individuals who had participated in a previous randomized, parallel-controlled trial (ClinicalTrials.gov number: NCT03614702) in Guangxi Province, China. The design of the previous randomized, parallel-controlled study has already been described in detail. Briefly, the eligible infants were randomized to receive one of six different sequential polio immunization schedules (sIPV-bOPV-bOPV, sIPV-sIPV-bOPV, sIPV-sIPV-tOPV, cIPV-bOPV-bOPV, cIPV-cIPV-bOPV, or cIPV-cIPV-tOPV; 200 participants per group). The three vaccine doses were administered at the ages of 2, 3, and 4 months old, respectively, and the antibody seroconversion rate was determined at 28 days after the third dose.

### Immune persistence study design

This extension study builds on the previous phase III study. At the ages of 24, 36, and 48 months, venous blood (2–3 ml) was collected from participants who had completed a basic three-dose polio immunization. The serum was separated from these blood samples and used for determining the neutralizing antibody titers against poliovirus types 1, 2, and 3. The Sabin strain are used as a challenge strain for testing of serum neutralizing antibody. The antibody-positive rate was determined; antibody positive was defined as having a neutralizing antibody titer of ≥1:8 against type 1, type 2, and type 3 poliovirus.

After each blood collection, a decision on whether to inoculate each participant with IPV to enhance their immunity depended on their previous neutralizing antibody results against poliovirus. The serum neutralizing antibody titer was measured 28 days after IPV inoculation.

All of the above neutralizing antibody titer tests were conducted by the National Institutes for Food and Drug Control, following the protocol recommended by the World Health Organization.

The present trial(ClinicalTrials.gov ID: NCT03821441) launched in 2018 and completed in 2020, was designed by the Institute of Medical Biology, Chinese Academy of Medical Sciences and the Guangxi Center for Disease Prevention and Control and were approved by the State Food and Drug Administration of China. The clinical trial protocol was verified and approved by the ethical committee of Guangxi Zhuang Autonomous Region for Disease Control and Prevention (approval number: GXIRB2017-0009-2) and was conducted in accordance with the Declaration of Helsinki (revised 2013). Informed consent was obtained in writing from the legal guardians of all study participants.

### Participants

The participants involved in the previous randomized, parallel-controlled study in Guangxi were recruited again. Eligible participants had completed a full course of primary vaccination against polio (three-dose immunization) and had a complete set of serum neutralizing antibody titer results available from the previous study. In addition, participants were required to be at least 24 months of age at the time of participating in the present clinical study. The exclusion criteria were as follows: (1) history of enhanced immunization with polio vaccine after completion of the previous clinical study; (2) history of confirmed infection with poliovirus; (3) participation in another study; and (4) another serious acute chronic disease or abnormality that could significantly increase the participant’s risk or interfere with the interpretation of our study results.

The guardians and families of the participants voluntarily complied with the requirements of the clinical trial protocol. Participants were permitted to voluntarily withdraw at any time during the trial. Participants could be withdrawn from the study in cases of failure to complete the follow-up visits, violation of or deviation from the trial protocol, or the appearance of other abnormal symptoms that interfered with the trial.

### Study objective

The primary objective of the present study was to evaluate the persistence of the poliovirus-specific antibody-positive rate in participants who received sequential immunization of IPV combined with bOPV.

### Statistical analysis

The seroconversion rates for antibody against poliovirus types 1, 2, and 3 were calculated in terms of each immunization schedule at 24, 36, and 48 months. Chi-squared tests and Fisher’s exact probability tests were used to compare the differences in seroconversion rates between groups.

The titers of antibodies against poliovirus types 1, 2, and 3 in all groups are presented as the geometric mean titers (GMTs) and their 95% confidence intervals. The differences in GMT between groups were compared by performing an analysis of variance after logarithmic transformation of the data. All statistical tests were considered significant at *p* < 0.05, two-sided.

Reverse cumulative distribution curves were plotted for the subsets of participants with effective antibody titers at 5 (i.e., 28 days after completion of basic immunization with three doses of vaccine), 24, 36, and 48 months of age.

### Reporting summary

Further information on research design is available in the Nature Research Reporting Summary linked to this article.

### Supplementary information

Supplement information

REPORTING SUMMARY

## Data Availability

The data that support the findings of this study are available from the corresponding author upon reasonable request.
