# Supplementary material for: Immune persistence after different polio sequential immunization schedules in Chinese infants
Source: NPJ Vaccines. 2024 Feb 29;9:50. doi: 10.1038/s41541-024-00831-1 (PMC10904800; doi:10.1038/s41541-024-00831-1)
Supplement: Supplementary file 1 — Supplement information [file 41541_2024_831_MOESM1_ESM.pdf]

Supplement table 1.the number of participants received supplementary IPV and enrolled the immunopersistence study

table1-1.the number of participants received supplementary IPV at 24, 36, 48 months

	sIPV-bOPV-bOPV	cIPV-bOPV-bOPV	cIPV-cIPV-bOPV	sIPV-sIPV-tOPV	sIPV-sIPV-bOPV	cIPV-cIPV-tOPV	Total
24 months	49	28	2	1	4	1	85
36 months	53	24	12	5	23	5	122
48 months	27	18	23	0	19	1	88

table1-2.the munber of participants enrolled the immunopersistence study at 24, 36, 48 months

	sIPV-bOPV-bOPV	cIPV-bOPV-bOPV	cIPV-cIPV-bOPV	sIPV-sIPV-tOPV	sIPV-sIPV-bOPV	cIPV-cIPV-tOPV	Total
24 months	173	170	159	163	169	166	1000
36 months	121	135	162	165	160	163	906
48 months	63	95	112	118	105	120	613

Supplement table 2.Multiple comparisons of seropositive rate against poliovirus (types 1, 2, and 3) 24,36 and 48 months after three doses of different immunization schedules

Months	Immunogenicity indicator	Group	Vs	Group	Test method	p-Value	Summary
24months	Type2	cIPV-bOPV-bOPV	Vs	sIPV-sIPV-tOPV	Pearson CHI-SQUARE	<0.0001	***
				sIPV-sIPV-bOPV	Pearson CHI-SQUARE	0.016	ns
				cIPV-cIPV-tOPV	Pearson CHI-SQUARE	<0.0001	***
				cIPV-cIPV-bOPV	Pearson CHI-SQUARE	<0.001	**
				sIPV-bOPV-bOPV	Pearson CHI-SQUARE	<0.0001	***
		sIPV-sIPV-tOPV	Vs	sIPV-sIPV-bOPV	Pearson CHI-SQUARE	<0.0001	***
				cIPV-cIPV-tOPV	Fisher exact test	1	ns
				cIPV-cIPV-bOPV	Pearson CHI-SQUARE	<0.0001	***
				sIPV-bOPV-bOPV	Pearson CHI-SQUARE	<0.0001	***
		sIPV-sIPV-bOPV	Vs	cIPV-cIPV-tOPV	Pearson CHI-SQUARE	<0.0001	***
				cIPV-cIPV-bOPV	Pearson CHI-SQUARE	0.19	ns
				sIPV-bOPV-bOPV	Pearson CHI-SQUARE	<0.0001	***
		cIPV-cIPV-tOPV	Vs	cIPV-cIPV-bOPV	Pearson CHI-SQUARE	<0.0001	***
				sIPV-bOPV-bOPV	Pearson CHI-SQUARE	<0.0001	***
		cIPV-cIPV-bOPV	Vs	sIPV-bOPV-bOPV	Pearson CHI-SQUARE	<0.0001	***
36months	Type2	cIPV-bOPV-bOPV	Vs	sIPV-sIPV-tOPV	Pearson CHI-SQUARE	<0.0001	***
				sIPV-sIPV-bOPV	Pearson CHI-SQUARE	0.07	ns

			cIPV-cIPV-tOPV	Pearson CHI-SQUARE	<0.0001	***
			cIPV-cIPV-bOPV	Pearson CHI-SQUARE	<0.001	**
			sIPV-bOPV-bOPV	Pearson CHI-SQUARE	<0.0001	***
	sIPV-sIPV-tOPV	Vs	sIPV-sIPV-bOPV	Pearson CHI-SQUARE	<0.0001	***
			cIPV-cIPV-tOPV	Fisher exact test	1	ns
			cIPV-cIPV-bOPV	Pearson CHI-SQUARE	<0.001	***
			sIPV-bOPV-bOPV	Pearson CHI-SQUARE	<0.0001	***
	sIPV-sIPV-bOPV	Vs	cIPV-cIPV-tOPV	Pearson CHI-SQUARE	<0.0001	***
			cIPV-cIPV-bOPV	Pearson CHI-SQUARE	0.12	ns
			sIPV-bOPV-bOPV	Pearson CHI-SQUARE	<0.0001	***
	cIPV-cIPV-tOPV	Vs	cIPV-cIPV-bOPV	Pearson CHI-SQUARE	<0.001	**
			sIPV-bOPV-bOPV	Pearson CHI-SQUARE	<0.0001	***
	cIPV-cIPV-bOPV	Vs	sIPV-bOPV-bOPV	Pearson CHI-SQUARE	<0.0001	***
Type3	cIPV-bOPV-bOPV	Vs	sIPV-sIPV-tOPV	Fisher exact test	0.034	ns
			sIPV-sIPV-bOPV	Fisher exact test	0.065	ns
			cIPV-cIPV-tOPV	Fisher exact test	0.034	ns
			cIPV-cIPV-bOPV	Fisher exact test	>0.99	ns
			sIPV-bOPV-bOPV	Fisher exact test	>0.99	ns
	sIPV-sIPV-tOPV	Vs	sIPV-sIPV-bOPV	Pearson CHI-SQUARE	1	ns
			cIPV-cIPV-tOPV	Pearson CHI-SQUARE	1	ns

				cIPV-cIPV-bOPV	Fisher exact test	0.03	ns
				sIPV-bOPV-bOPV	Fisher exact test	0.04	ns
		sIPV-sIPV-bOPV	Vs	cIPV-cIPV-tOPV	Pearson CHI-SQUARE	1	ns
				cIPV-cIPV-bOPV	Fisher exact test	0.29	ns
				sIPV-bOPV-bOPV	Fisher exact test	0.07	ns
		cIPV-cIPV-tOPV	Vs	cIPV-cIPV-bOPV	Fisher exact test	0.03	ns
				sIPV-bOPV-bOPV	Fisher exact test	0.04	ns
		cIPV-cIPV-bOPV	Vs	sIPV-bOPV-bOPV	Fisher exact test	>0.99	ns
48months	Type2	cIPV-bOPV-bOPV	Vs	sIPV-sIPV-tOPV	Pearson CHI-SQUARE	<0.0001	***
				sIPV-sIPV-bOPV	Pearson CHI-SQUARE	0.86	ns
				cIPV-cIPV-tOPV	Pearson CHI-SQUARE	<0.0001	***
				cIPV-cIPV-bOPV	Pearson CHI-SQUARE	0.24	ns
				sIPV-bOPV-bOPV	Pearson CHI-SQUARE	0.002	*
		sIPV-sIPV-tOPV	Vs	sIPV-sIPV-bOPV	Pearson CHI-SQUARE	<0.0001	***
				cIPV-cIPV-tOPV	Fisher exact test	1	ns
				cIPV-cIPV-bOPV	Pearson CHI-SQUARE	<0.0001	***
				sIPV-bOPV-bOPV	Pearson CHI-SQUARE	<0.0001	***
		sIPV-sIPV-bOPV	Vs	cIPV-cIPV-tOPV	Pearson CHI-SQUARE	<0.0001	***
				cIPV-cIPV-bOPV	Pearson CHI-SQUARE	0.14	ns
				sIPV-bOPV-bOPV	Pearson CHI-SQUARE	0.002	*

cIPV-cIPV-tOPV	Vs	cIPV-cIPV-bOPV	Pearson CHI-SQUARE	<0.001	**
		sIPV-bOPV-bOPV	Pearson CHI-SQUARE	<0.0001	***
cIPV-cIPV-bOPV	Vs	sIPV-bOPV-bOPV	Pearson CHI-SQUARE	<0.0001	***

We assessed the statistical significance of pairwise comparisons based on Bonferroni'adjustment α levels. The α level was adjusted to 0.008333, that is, the ratio of the original α level (0.05) to the number of groups (6).

Supplement table 3.Multiple comparisons of GMT against poliovirus (types 1, 2, and 3) 24,36 and 48 months after three doses of different immunization schedules

table3-1.Multiple comparisons of GMT against poliovirus type 1 at 24 month after three doses of different immunization schedules

Tukey's multiple comparisons test	Mean Diff.	95.00% CI of diff.	Significant	Summary	Adjusted P Value
cIPV-bOPV-bOPV vs. sIPV-sIPV-bOPV	-0.1295	-0.2901 to 0.03119	No	ns	0.1946
cIPV-bOPV-bOPV vs. cIPV-cIPV-bOPV	0.1253	-0.03783 to 0.2885	No	ns	0.2417
cIPV-bOPV-bOPV vs. sIPV-sIPV-tOPV	0.1058	-0.05635 to 0.2679	No	ns	0.426
cIPV-bOPV-bOPV vs. cIPV-cIPV-tOPV	0.3334	0.1721 to 0.4948	Yes	****	<0.0001
cIPV-bOPV-bOPV vs. sIPV-bOPV-bOPV	-0.191	-0.3507 to -0.0313	Yes	**	0.0087
sIPV-sIPV-bOPV vs. cIPV-cIPV-bOPV	0.2548	0.09139 to 0.4182	Yes	***	0.0001
sIPV-sIPV-bOPV vs. sIPV-sIPV-tOPV	0.2352	0.07286 to 0.3976	Yes	***	0.0005
sIPV-sIPV-bOPV vs. cIPV-cIPV-tOPV	0.4629	0.3013 to 0.6245	Yes	****	<0.0001
sIPV-sIPV-bOPV vs. sIPV-bOPV-bOPV	-0.06156	-0.2215 to 0.09839	No	ns	0.8819
cIPV-cIPV-bOPV vs. sIPV-sIPV-tOPV	-0.01956	-0.1844 to 0.1453	No	ns	0.9994
cIPV-cIPV-bOPV vs. cIPV-cIPV-tOPV	0.2081	0.04401 to 0.3722	Yes	**	0.0042
cIPV-cIPV-bOPV vs. sIPV-bOPV-bOPV	-0.3163	-0.4788 to -0.1539	Yes	****	<0.0001
sIPV-sIPV-tOPV vs. cIPV-cIPV-tOPV	0.2277	0.06461 to 0.3907	Yes	**	0.001
sIPV-sIPV-tOPV vs. sIPV-bOPV-bOPV	-0.2968	-0.4582 to -0.1353	Yes	****	<0.0001
cIPV-cIPV-tOPV vs. sIPV-bOPV-bOPV	-0.5244	-0.6851 to -0.3638	Yes	****	<0.0001

table3-2. Multiple comparisons of GMT against poliovirus type 2 at 24 month after three doses of different immunization schedules

Dunn's multiple comparisons test	Mean rank diff.	Significant	Summary	Adjusted P Value
cIPV-bOPV-bOPV vs. sIPV-sIPV-bOPV	-74.58	No	ns	0.253
cIPV-bOPV-bOPV vs. cIPV-cIPV-bOPV	-101.2	Yes	*	0.0212
cIPV-bOPV-bOPV vs. sIPV-sIPV-tOPV	-437.4	Yes	****	<0.0001
cIPV-bOPV-bOPV vs. cIPV-cIPV-tOPV	-429.5	Yes	****	<0.0001
cIPV-bOPV-bOPV vs. sIPV-bOPV-bOPV	159.5	Yes	****	<0.0001
sIPV-sIPV-bOPV vs. cIPV-cIPV-bOPV	-26.6	No	ns	>0.9999
sIPV-sIPV-bOPV vs. sIPV-sIPV-tOPV	-362.8	Yes	****	<0.0001
sIPV-sIPV-bOPV vs. cIPV-cIPV-tOPV	-354.9	Yes	****	<0.0001
sIPV-sIPV-bOPV vs. sIPV-bOPV-bOPV	234	Yes	****	<0.0001
cIPV-cIPV-bOPV vs. sIPV-sIPV-tOPV	-336.2	Yes	****	<0.0001
cIPV-cIPV-bOPV vs. cIPV-cIPV-tOPV	-328.3	Yes	****	<0.0001
cIPV-cIPV-bOPV vs. sIPV-bOPV-bOPV	260.6	Yes	****	<0.0001
sIPV-sIPV-tOPV vs. cIPV-cIPV-tOPV	7.974	No	ns	>0.9999
sIPV-sIPV-tOPV vs. sIPV-bOPV-bOPV	596.9	Yes	****	<0.0001
cIPV-cIPV-tOPV vs. sIPV-bOPV-bOPV	588.9	Yes	****	<0.0001

table3-3. Multiple comparisons of GMT against poliovirus type 3 at 24 month after three doses of different immunization schedules

Dunn's multiple comparisons test	Mean rank diff.	Significant	Summary	Adjusted P Value
cIPV-bOPV-bOPV vs. sIPV-sIPV-bOPV	-93.81	Yes	*	0.0402
cIPV-bOPV-bOPV vs. cIPV-cIPV-bOPV	-80.98	No	ns	0.1609
cIPV-bOPV-bOPV vs. sIPV-sIPV-tOPV	-7.843	No	ns	>0.9999
cIPV-bOPV-bOPV vs. cIPV-cIPV-tOPV	1.224	No	ns	>0.9999
cIPV-bOPV-bOPV vs. sIPV-bOPV-bOPV	-5.806	No	ns	>0.9999
sIPV-sIPV-bOPV vs. cIPV-cIPV-bOPV	12.83	No	ns	>0.9999
sIPV-sIPV-bOPV vs. sIPV-sIPV-tOPV	85.97	No	ns	0.0973
sIPV-sIPV-bOPV vs. cIPV-cIPV-tOPV	95.03	Yes	*	0.0375
sIPV-sIPV-bOPV vs. sIPV-bOPV-bOPV	88	No	ns	0.0702
cIPV-cIPV-bOPV vs. sIPV-sIPV-tOPV	73.14	No	ns	0.3384
cIPV-cIPV-bOPV vs. cIPV-cIPV-tOPV	82.2	No	ns	0.1503
cIPV-cIPV-bOPV vs. sIPV-bOPV-bOPV	75.17	No	ns	0.2607
sIPV-sIPV-tOPV vs. cIPV-cIPV-tOPV	9.067	No	ns	>0.9999
sIPV-sIPV-tOPV vs. sIPV-bOPV-bOPV	2.037	No	ns	>0.9999
cIPV-cIPV-tOPV vs. sIPV-bOPV-bOPV	-7.03	No	ns	>0.9999

table3-4. Multiple comparisons of GMT against poliovirus type 1 at 36 month after three doses of different immunization schedules

Dunn's multiple comparisons test	Mean rank diff.	Significant	Summary	Adjusted P Value
cIPV-bOPV-bOPV vs. sIPV-sIPV-bOPV	-103.8	Yes	**	0.0099
cIPV-bOPV-bOPV vs. cIPV-cIPV-bOPV	31.81	No	ns	>0.9999
cIPV-bOPV-bOPV vs. sIPV-sIPV-tOPV	14.54	No	ns	>0.9999
cIPV-bOPV-bOPV vs. cIPV-cIPV-tOPV	147.9	Yes	****	<0.0001
cIPV-bOPV-bOPV vs. sIPV-bOPV-bOPV	-104.6	Yes	*	0.0204
sIPV-sIPV-bOPV vs. cIPV-cIPV-bOPV	135.6	Yes	****	<0.0001
sIPV-sIPV-bOPV vs. sIPV-sIPV-tOPV	118.3	Yes	***	0.0006
sIPV-sIPV-bOPV vs. cIPV-cIPV-tOPV	251.6	Yes	****	<0.0001
sIPV-sIPV-bOPV vs. sIPV-bOPV-bOPV	-0.7671	No	ns	>0.9999
cIPV-cIPV-bOPV vs. sIPV-sIPV-tOPV	-17.28	No	ns	>0.9999
cIPV-cIPV-bOPV vs. cIPV-cIPV-tOPV	116	Yes	***	0.0009
cIPV-cIPV-bOPV vs. sIPV-bOPV-bOPV	-136.4	Yes	***	0.0002
sIPV-sIPV-tOPV vs. cIPV-cIPV-tOPV	133.3	Yes	****	<0.0001
sIPV-sIPV-tOPV vs. sIPV-bOPV-bOPV	-119.1	Yes	**	0.002
cIPV-cIPV-tOPV vs. sIPV-bOPV-bOPV	-252.4	Yes	****	<0.0001

table3-5. Multiple comparisons of GMT against poliovirus type 2 at 36 month after three doses of different immunization schedules

Dunn's multiple comparisons test	Mean rank diff.	Significant	Summary	Adjusted P Value
cIPV-bOPV-bOPV vs. sIPV-sIPV-bOPV	-50.9	No	ns	>0.9999
cIPV-bOPV-bOPV vs. cIPV-cIPV-bOPV	-80.55	No	ns	0.1206
cIPV-bOPV-bOPV vs. sIPV-sIPV-tOPV	-389	Yes	****	<0.0001
cIPV-bOPV-bOPV vs. cIPV-cIPV-tOPV	-381.8	Yes	****	<0.0001
cIPV-bOPV-bOPV vs. sIPV-bOPV-bOPV	139.7	Yes	***	0.0003
sIPV-sIPV-bOPV vs. cIPV-cIPV-bOPV	-29.65	No	ns	>0.9999
sIPV-sIPV-bOPV vs. sIPV-sIPV-tOPV	-338	Yes	****	<0.0001
sIPV-sIPV-bOPV vs. cIPV-cIPV-tOPV	-330.9	Yes	****	<0.0001
sIPV-sIPV-bOPV vs. sIPV-bOPV-bOPV	190.6	Yes	****	<0.0001
cIPV-cIPV-bOPV vs. sIPV-sIPV-tOPV	-308.4	Yes	****	<0.0001
cIPV-cIPV-bOPV vs. cIPV-cIPV-tOPV	-301.3	Yes	****	<0.0001
cIPV-cIPV-bOPV vs. sIPV-bOPV-bOPV	220.3	Yes	****	<0.0001
sIPV-sIPV-tOPV vs. cIPV-cIPV-tOPV	7.102	No	ns	>0.9999
sIPV-sIPV-tOPV vs. sIPV-bOPV-bOPV	528.7	Yes	****	<0.0001
cIPV-cIPV-tOPV vs. sIPV-bOPV-bOPV	521.6	Yes	****	<0.0001

table3-6. Multiple comparisons of GMT against poliovirus type 3 at 36 month after three doses of different immunization schedules

Dunn's multiple comparisons test	Mean rank diff.	Significant	Summary	Adjusted P Value
cIPV-bOPV-bOPV vs. sIPV-sIPV-bOPV	-85.64	No	ns	0.074
cIPV-bOPV-bOPV vs. cIPV-cIPV-bOPV	-67.76	No	ns	0.3857
cIPV-bOPV-bOPV vs. sIPV-sIPV-tOPV	8.02	No	ns	>0.9999
cIPV-bOPV-bOPV vs. cIPV-cIPV-tOPV	29.71	No	ns	>0.9999
cIPV-bOPV-bOPV vs. sIPV-bOPV-bOPV	19.57	No	ns	>0.9999
sIPV-sIPV-bOPV vs. cIPV-cIPV-bOPV	17.88	No	ns	>0.9999
sIPV-sIPV-bOPV vs. sIPV-sIPV-tOPV	93.66	Yes	*	0.018
sIPV-sIPV-bOPV vs. cIPV-cIPV-tOPV	115.3	Yes	**	0.0011
sIPV-sIPV-bOPV vs. sIPV-bOPV-bOPV	105.2	Yes	*	0.0121
cIPV-cIPV-bOPV vs. sIPV-sIPV-tOPV	75.78	No	ns	0.1287
cIPV-cIPV-bOPV vs. cIPV-cIPV-tOPV	97.47	Yes	*	0.0113
cIPV-cIPV-bOPV vs. sIPV-bOPV-bOPV	87.33	No	ns	0.0795
sIPV-sIPV-tOPV vs. cIPV-cIPV-tOPV	21.69	No	ns	>0.9999
sIPV-sIPV-tOPV vs. sIPV-bOPV-bOPV	11.55	No	ns	>0.9999
cIPV-cIPV-tOPV vs. sIPV-bOPV-bOPV	-10.14	No	ns	>0.9999

table3-7. Multiple comparisons of GMT against poliovirus type 1 at 48 month after three doses of different immunization schedules

Tukey's multiple comparisons test	Mean Diff.	95.00% CI of diff.	Significant	Summary	Adjusted P Value
cIPV-bOPV-bOPV vs. sIPV-sIPV-bOPV	-0.1534	-0.3636 to 0.05682	No	ns	0.2959
cIPV-bOPV-bOPV vs. cIPV-cIPV-bOPV	0.1264	-0.08072 to 0.3334	No	ns	0.5028
cIPV-bOPV-bOPV vs. sIPV-sIPV-tOPV	0.04055	-0.1641 to 0.2452	No	ns	0.9931
cIPV-bOPV-bOPV vs. cIPV-cIPV-tOPV	0.3378	0.1339 to 0.5417	Yes	****	<0.0001
cIPV-bOPV-bOPV vs. sIPV-bOPV-bOPV	-0.3346	-0.5758 to -0.09338	Yes	**	0.0011
sIPV-sIPV-bOPV vs. cIPV-cIPV-bOPV	0.2798	0.07809 to 0.4814	Yes	**	0.0011
sIPV-sIPV-bOPV vs. sIPV-sIPV-tOPV	0.194	-0.005225 to 0.3931	No	ns	0.0614
sIPV-sIPV-bOPV vs. cIPV-cIPV-tOPV	0.4912	0.2928 to 0.6896	Yes	****	<0.0001
sIPV-sIPV-bOPV vs. sIPV-bOPV-bOPV	-0.1812	-0.4178 to 0.05541	No	ns	0.2442
cIPV-cIPV-bOPV vs. sIPV-sIPV-tOPV	-0.08582	-0.2817 to 0.11	No	ns	0.8105
cIPV-cIPV-bOPV vs. cIPV-cIPV-tOPV	0.2114	0.01636 to 0.4065	Yes	*	0.0248
cIPV-cIPV-bOPV vs. sIPV-bOPV-bOPV	-0.461	-0.6948 to -0.2272	Yes	****	<0.0001
sIPV-sIPV-tOPV vs. cIPV-cIPV-tOPV	0.2972	0.1048 to 0.4897	Yes	***	0.0002
sIPV-sIPV-tOPV vs. sIPV-bOPV-bOPV	-0.3752	-0.6068 to -0.1435	Yes	****	<0.0001
cIPV-cIPV-tOPV vs. sIPV-bOPV-bOPV	-0.6724	-0.9034 to -0.4414	Yes	****	<0.0001

table3-8. Multiple comparisons of GMT against poliovirus type 2 at 48 month after three doses of different immunization schedules

Dunn's multiple comparisons test	Mean rank diff.	Significant	Summary	Adjusted P Value
cIPV-bOPV-bOPV vs. sIPV-sIPV-bOPV	-10.35	No	ns	>0.9999
cIPV-bOPV-bOPV vs. cIPV-cIPV-bOPV	-30.99	No	ns	>0.9999
cIPV-bOPV-bOPV vs. sIPV-sIPV-tOPV	-253.1	Yes	****	<0.0001
cIPV-bOPV-bOPV vs. cIPV-cIPV-tOPV	-251.7	Yes	****	<0.0001
cIPV-bOPV-bOPV vs. sIPV-bOPV-bOPV	57.96	No	ns	0.6508
sIPV-sIPV-bOPV vs. cIPV-cIPV-bOPV	-20.64	No	ns	>0.9999
sIPV-sIPV-bOPV vs. sIPV-sIPV-tOPV	-242.8	Yes	****	<0.0001
sIPV-sIPV-bOPV vs. cIPV-cIPV-tOPV	-241.4	Yes	****	<0.0001
sIPV-sIPV-bOPV vs. sIPV-bOPV-bOPV	68.31	No	ns	0.2282
cIPV-cIPV-bOPV vs. sIPV-sIPV-tOPV	-222.1	Yes	****	<0.0001
cIPV-cIPV-bOPV vs. cIPV-cIPV-tOPV	-220.7	Yes	****	<0.0001
cIPV-cIPV-bOPV vs. sIPV-bOPV-bOPV	88.95	Yes	*	0.0207
sIPV-sIPV-tOPV vs. cIPV-cIPV-tOPV	1.398	No	ns	>0.9999
sIPV-sIPV-tOPV vs. sIPV-bOPV-bOPV	311.1	Yes	****	<0.0001
cIPV-cIPV-tOPV vs. sIPV-bOPV-bOPV	309.7	Yes	****	<0.0001

table3-9. Multiple comparisons of GMT against poliovirus type 3 at 48 month after three doses of different immunization schedules

Dunn's multiple comparisons test	Mean rank diff.	Significant	Summary	Adjusted P Value
cIPV-bOPV-bOPV vs. sIPV-sIPV-bOPV	-69.91	No	ns	0.0766
cIPV-bOPV-bOPV vs. cIPV-cIPV-bOPV	-54.77	No	ns	0.3892
cIPV-bOPV-bOPV vs. sIPV-sIPV-tOPV	1.784	No	ns	>0.9999
cIPV-bOPV-bOPV vs. cIPV-cIPV-tOPV	16.68	No	ns	>0.9999
cIPV-bOPV-bOPV vs. sIPV-bOPV-bOPV	-42.69	No	ns	>0.9999
sIPV-sIPV-bOPV vs. cIPV-cIPV-bOPV	15.14	No	ns	>0.9999
sIPV-sIPV-bOPV vs. sIPV-sIPV-tOPV	71.69	Yes	*	0.0366
sIPV-sIPV-bOPV vs. cIPV-cIPV-tOPV	86.59	Yes	**	0.0036
sIPV-sIPV-bOPV vs. sIPV-bOPV-bOPV	27.22	No	ns	>0.9999
cIPV-cIPV-bOPV vs. sIPV-sIPV-tOPV	56.55	No	ns	0.2256
cIPV-cIPV-bOPV vs. cIPV-cIPV-tOPV	71.45	Yes	*	0.0306
cIPV-cIPV-bOPV vs. sIPV-bOPV-bOPV	12.08	No	ns	>0.9999
sIPV-sIPV-tOPV vs. cIPV-cIPV-tOPV	14.9	No	ns	>0.9999
sIPV-sIPV-tOPV vs. sIPV-bOPV-bOPV	-44.47	No	ns	>0.9999
cIPV-cIPV-tOPV vs. sIPV-bOPV-bOPV	-59.37	No	ns	0.4565

Supplement table 4.Seropositive rate and geometric mean titer against poliovirus types 1, 2, and 3 included in the set of infants who had antibody titers determined for blood samples collected at 24,36 and 48 months of age

	sIPV-bOPV-bOPV (n=44)	cIPV-bOPV-bOPV (n=75)	cIPV-cIPV-bOPV (n=88)	sIPV-sIPV-tOPV (n=103)	sIPV-sIPV-bOPV (n=88)	cIPV-cIPV-tOPV (n=98)	*P* value	Test method
**24 months**								
**Type 1**								
**Seropositive, n (%)**	44(100,92-100)	75(100,95.1-100)	88(100,95.8-100)	103(100,96.4-100)	88(100,95.8-100)	98(100,96.2-100)	1	Fisher exact
**GMT (95% CI)**	1026(769.2-1368)	743.7(590.8-936.2)	490.3(382.6-628.2)	626.2(493-795.2)	981.3(768.7-1253)	281.1(220.8-358)	<0.0001	ANOVA
**Type 2**								
**Seropositive, n (%)**	29(65.9,51.1-78.1)	59(90.8,81.3-95.7)	86(97.7,92.1-99.6)	103(100,96.4-100)	81(92,84.5-96.1)	98(100,96.2-100)	<0.0001	Pearson Chi-Square
**GMT (95% CI)**	12.8(9-18.1)	30.31(22.82-40.27)	40.9(32.3-51.9)	298.9(246-363.1)	36.75(28.84-46.82)	273.1(221.4-337)	<0.0001	ANOVA
**Type 3**								
**Seropositive, n (%)**	44(100,92-100)	75(100,95.1-100)	88(100,95.8-100)	103(100,96.4-100)	88(100,95.8-100)	98(100,96.2-100)	1	Fisher exact
**GMT (95% CI)**	252.5(183.1-348.1)	255.6(201.8-323.6)	337.5(266.9-426.7)	235.3(183-302.6)	369.7(299.1-457)	195.2(148.3-257)	0.002	ANOVA
**36 months**								
**Type 1**								
**Seropositive, n (%)**	44(100,92-100)	75(100,95.1-100)	87(98.9,93.8-99.9)	103(100,96.4-100)	88(100,95.8-100)	98(100,96.2-100)	0.6	Fisher exact
**GMT (95% CI)**	1005(736.1-1371)	603.1(487-746.8)	435.6(333.5-569.1)	536.4(421-683.5)	920(724.7-1168)	261.8(204-336)	<0.0001	ANOVA
**Type 2**								
**Seropositive, n (%)**	27(61.4,46.6-74.3)	66(88,78.7-93.6)	82(93.2,85.9-96.8)	103(100,96.4-100)	79(89.8,81.7-94.5)	98(100,96.2-100)	<0.0001	Pearson Chi-Square
**GMT (95% CI)**	15(9.6-23.5)	31.4(23.2-42.6)	41.1(31.9-53)	337.1(278.7-407.8)	40.8(31.2-53.3)	314.7(254.9-388.4)	<0.0001	ANOVA
**Type 3**								
**Seropositive, n (%)**	44(100,92-100)	75(100,95.1-100)	88(100,95.8-100)	101(98.1,93.2-99.7)	88(100,95.8-100)	97(99,94.4-99.9)	0.64	Fisher exact
**GMT (95% CI)**	251.5(167.1-378.6)	224.7(177.5-284.5)	268.2(216.3-332.5)	186.8(144.7-241.2)	333.2(268.1-414.2)	170(128.8-224.4)	0.0017	ANOVA

**48 months**								
**Type 1**								
**Seropositive, n (%)**	44(100,92-100)	75(100,95.1-100)	87(98.9,93.8-99.9)	103(100,96.4-100)	88(100,95.8-100)	97(99,94.4-99.9)	0.77	Fisher exact
**GMT (95% CI)**	1165(874.5-1553)	561(441-713.7)	401.8(308.6-523.1)	547(429.3-697)	797.9(612-1040)	275.9(210.5-361.7)	<0.0001	ANOVA
**Type 2**								
**Seropositive, n (%)**	29(65.9,51.1-78.1)	64(85.3,75.6-91.6)	80(90.9,83.1-95.3)	103(100,96.4-100)	72(81.8,72.5-88.5)	97(99,94.4-99.9)	<0.0001	Pearson Chi-Square
**GMT (95% CI)**	14.2(9.8-20.5)	24(17.8-32.5)	29.2(22.8-37.3)	185.3(153.1-224.4)	23.7(18.6-30.1)	181.6(147.2-223.9)	<0.0001	ANOVA
**Type 3**								
**Seropositive, n (%)**	44(100,92-100)	75(100,95.1-100)	88(100,95.8-100)	102(99,94.7-100)	87(98.9,93.8-99.9)	98(100,96.2-100)	0.92	Fisher exact
**GMT (95% CI)**	292.5(199.1-429.5)	212.1(162.3-277.1)	274.6(219.4-343.7)	189.1(146.6-244)	285.4(230.9-352.9)	160.2(124.4-206.3)	0.0024	ANOVA

ClinicalTrials.gov PRS DRAFT Receipt (Working Version)
Last Update: 08/10/2018 04:05

ClinicalTrials.gov ID: [Not yet assigned]

Study Identification

Unique Protocol ID: 201518502-C (bOPV-PRO-C)

Brief Title: Trail To Evaluate the Immune Effects of Primary and Booster Immunizations With Poliovirus Vaccine

Official Title: Trail To Evaluate the Immunity Duration of Different Sequential Immunization Schedules and Effectiveness for Bivalent Oral Poliomyelitis Vaccine Co-administered With IPV Booster Immunization for Poliovirus Vaccine

Secondary IDs:

Study Status

Record Verification: May 2018

Overall Status: Recruiting

Study Start: January 4, 2018 [Actual]

Primary Completion: May 2020 [Anticipated]

Study Completion: August 2020 [Anticipated]

Sponsor/Collaborators

Sponsor: Chinese Academy of Medical Sciences

Responsible Party: Principal Investigator
Investigator: Jingsi Yang [jiyang]
Official Title: Senior
Affiliation: Chinese Academy of Medical Sciences

Collaborators: Guangxi Center for Disease Control and Prevention

Oversight

U.S. FDA-regulated Drug: No

U.S. FDA-regulated Device: No

U.S. FDA IND/IDE: No

Human Subjects Review: Board Status: Approved
Approval Number: GXIRB2017-0009-2
Board Name: GUANXI IRB
Board Affiliation: Guangxi Province Centers for Disease Control and Prevention
Phone: +867712518979
Email: mozhj@126.com
Address:

18# Jinzhou Road, Nanning, Guangxi Province

Data Monitoring: Yes

FDA Regulated Intervention: No

Study Description

Brief Summary: Trail To Evaluate the Immunity Duration of healthy children who already took part in " The safety and immunogenicity by different sequential schedules of bOPV and bOPV in dragee candy with sIPV, a randomized, double blind, single center and parallel phase III clinic trial was performed in Infants of two-month old in Guangxi Province, China" and continue to search for the effects of booster immunization.

Detailed Description: According to the requirement of the Strategy of Polio Eradication & Endgame Strategic Plan 2013-2018, bivalent oral attenuated live poliomyelitis vaccine against type 1 and 3 (bOPV) and inactivated poliomyelitis vaccine made by Sabin strain (sIPV) need to be used to eradiation both the wild poliovirus and vaccine-derived poliovirus. To evaluate the safety and immunogenicity by different sequential immunization schedules of bOPV and bOPV in dragee candy with sIPV,a randomized, double blind, single center and parallel phase III clinic trial was performed in Guangxi Province in China. A total of 1200 infants at 2 months old were selected, and randomlydivided into 12 different groups (100 individuals were included in each group) administrated the vaccines at 0, 28, 56 days schedule.The detail of each group as following:1)1-dose cIPV + 2-dose bOPV (Candy); 2)1-dose sIPV + 2-dose bOPV (Candy); 3)2-dose cIPV + 1-dose bOPV (Candy); 4)2-dose sIPV + 1-dose bOPV (Candy); 5)2-dose cIPV + 1-dose tOPV (Candy); 6)2-dose sIPV + 1-dose tOPV (Candy); 7)1-dose cIPV + 2-dose bOPV (Liquid); 8)1-dose sIPV + 2-dose bOPV (Liquid); 9)2-dose cIPV + 1-dose bOPV (Liquid); 10)2-dose sIPV + 1-dose bOPV (Liquid); 11)2-dose cIPV + 1-dose tOPV (Liquid); 12)2-dose sIPV + 1-dose tOPV (Liquid).Blood Sample was collected before vaccination and one month after the third dose of vaccination. Neutralization antibody against type I, Type I and Type III poliomyelitis virus were detected to evaluate the seroprotection rates and antibody geometric mean concentrations. The fecal samples were collected to test viral shedding.The safety by different sequential schedule of the vaccines was also evaluated.This part of study have already been done in 2016.

To further evaluate the immunity duration of different sequential immunization schedules for bOPV and IPV ,more importantly,trying to research the effectiveness of bOPV booster immunization,the previous study will continue.

The detail of the research as following:

The subject will been recruited again,all subjects have already took part in phase 3 clinical trail in Guangxi ,vaccinated 3-dose primary immunization with polio vaccines and with the result of the paired serum .In order to evaluate the immune effects of primary immunizations with poliovirus vaccine(3 doses of immunization). Blood samples were collected when the subject aged 24 months old,36 months old and 48 months old. Neutralization antibody against type I, Type I and Type III poliomyelitis virus were detected to evaluate the positive rate and antibody geometric mean titers.

In order to research the effectiveness of bOPV booster immunization, when subjects aged 48 months,they should take 1-dose bOPV(liquid/candy) as boosting immunization.The vaccine producer and vaccine dosage form of bOPV should same with primary vaccination .If bOPV(candy/liquid) is not available ,in order to protect the rights and interests of subjects ,the investigator can adjust other poliovirus vaccine such as IPV replace bOPV.

The anticoagulant blood collected from subjects aged 48 months and the 28 days after booster immunization will been used to detect cellular immunity situation.

Conditions

Conditions: Poliomyelitis

Keywords: bOPV, IPV, Immunity Duration,Booster Immunization

Study Design

Study Type: Interventional

Primary Purpose: Prevention

Study Phase: Phase 3

Interventional Study Model: Parallel Assignment

Number of Arms: 3

Masking: Quadruple (Participant, Care Provider, Investigator, Outcomes Assessor)

Allocation: Non-Randomized

Enrollment: 1165 [Anticipated]

Arms and Interventions

Arms	Assigned Interventions
Experimental: bOPV(Candy)<br>bOPV (Candy): bivalent oral attenuated live poliomyelitis vaccine against type 1 and type 3 in Dragee Candy (Human Diploid Cell) Produced by Institute of Medical Biology, Chinese Academy of Medical Sciences.<br>1g/pill,10 pills/pach,one pill each time; each pill containing polio virus≥5.92 lgCCID50 including type1 polio virus≥5.8 lgCCID50，type 3 polio virus ≥5.3lgCCID50.	Biological/Vaccine: bOPV(Candy)<br>A single dose of 1 pill orally of bOPV to subjects aged 48 months who recieved bOPV (candy) produced by institute of Medical Biology, Chinese Academy of Medical Sciences in primary immunization. If bOPV(candy/liquid) is not available ,in order to protect the rights and interests of subjects ,the investigator can adjust other poliovirus vaccine such as IPV replace bOPV.
Experimental: bOPV(Liquid)<br>bOPV (Liquid): bivalent oral attenuated live poliomyelitis vaccine against type 1 and type 3 (Human Diploid Cell) Produced by Institute of Medical Biology, Chinese Academy of Medical Sciences.<br>0.5ml or 1.0ml each bottle;total content of polio virus≥7.12lgCCID50/ml，type1 polio virus≥7.0 lgCCID50/ml，type 3 polio virus ≥6.5lgCCID50/ml. (2 drops each person;be be equivalent to 0.1ml each person)	Biological/Vaccine: bOPV(Liquid)<br>A single dose of 2 drops (0.1 ml) orally of bOPV to subjects aged 48 months who recieved bOPV (liquid) produced by institute of Medical Biology, Chinese Academy of Medical Sciences in primary immunization. If bOPV(candy/liquid) is not available,in order to protect the rights and interests of subjects ,the investigator can adjust other poliovirus vaccine such as IPV replace bOPV.
Experimental: bOPV（liquid）<br>bOPV(Liquid):Poliomyelitis (Live) Vaccine Type I Type III (Human Diploid Cell), Oral Produced by Beijing Tiantan Biological Products Co., Ltd. 1.0ml each bottle,2 drops each person(be be equivalent to 0.1ml each person).(total content of polio virus≥6.12lgCCID50，type1 polio virus≥6.0	Biological/Vaccine: bOPV(Liquid)<br>A single dose of 2 drops (0.1 ml) orally of bOPV to subjects aged 48 months who received tOPV (liquid) produced by Beijing Tiantan Biological Products Co., Ltd. in primary immunization. If bOPV(candy/liquid) is not available,in order to protect the rights and interests

Arms	Assigned Interventions
lgCCID50，type 3 polio virus ≥5.5lgCCID50 in each 0.1 ml)	of subjects ,the investigator can adjust other poliovirus vaccine such as IPV replace bOPV.

NOTE : More than one Intervention has the name 'bOPV(Liquid)'

Outcome Measures

Primary Outcome Measure:

1. Evaluate the effectiveness bOPV(candy/liquid) co-administered with IPV.
   To evaluate the effectiveness of bivalent oral attenuated live poliomyelitis vaccine against type 1 and type 3 (Human Diploid Cell) (candy/liquid) co-administered with IPV by neutralization assay. Defined ≥ 8 (1/dil) for anti-Poliovirus as positive.

   [Time Frame: at aged 24、36、48 months]

Secondary Outcome Measure:

2. The duration of anti-poliovirus antibodies level.
   Using the neutralization assay to research the duration of anti-poliovirus antibodies types1, 2, and 3 of bivalent oral attenuated live poliomyelitis vaccine against type 1 and type 3 (Human Diploid Cell) (candy/liquid) co-administered with IPV.

   [Time Frame: at aged 24、36、48 months]
3. The adverse reaction and event of EV71 vaccine occur in subjects
   Local and systemic adverse events were active collected in subjects after boosting dose of bOPV.

   [Time Frame: following 28 days after the boosting dose of bOPV]
4. Rationality of booster immunization
   Give one dose boosting vaccine (bOPV) when the subject aged 48 months.The type of bOPV (liquid/candy) depends on what kind of vaccine the child had eaten in" Randomized, Double Blind, Single Center, Parallel Trial to Evaluate the Safety and Immunogenicity by Different Sequential Immunization Schedules of Bivalent Oral Poliomyelitis Vaccine Co-administered With IPV in Infants Aged 2 Months."

   [Time Frame: at aged 48 months and 28 days after the boosting dose of bOPV]

Eligibility

Minimum Age: 24 Months

Maximum Age: 48 Months

Sex: All

Gender Based: No

Accepts Healthy Volunteers: Yes

Criteria: Inclusion Criteria:

- Subjects who have already took part in phase 3 clinical trail in Guangxi and was vaccinated 3-dose primary immunization with polio vaccines .Moreover，the results of the selected paired serum are required.
- 24 months old(calendar month).
- Guardians understand the contents and requirements of this trail , meanwhile, voluntarily joined this study with informed consents.
- Able to attend all scheduled visits and to comply with all trial procedures(including vaccinate and blood collection)

Exclusion Criteria:

- Any booster immunization with polio vaccine after finishing 3-dose primary immunizations research.
- Polio virus infection was demonstrated in laboratory experiment.
- Participation in another clinical trial at the same times.
- Any condition that in the opinion of the investigator, may interfere with the evaluation of study objectives or increase the risk of subjects,such as acute or chronic diseases 、 some abnormal detected by lab,and so on.

Contacts/Locations

Central Contact Person: Jingsi Yang, Master
Telephone: +8687168334986
Email: yjs@imbcams.com.cn

Central Contact Backup: Jing Li, Master
Telephone: 13888865251
Email: sola@imbcams.com.cn

Study Officials: Zhaojun Mo, Master
Study Principal Investigator
Guangxi Province Center for Diseases Control and Prevention

Locations: China, Guangxi
Guangxi Provincial Center for Diseases Control and Prevention
[Recruiting]
Nanning, Guangxi, China
Contact: Zhaojun Mo, Master
Principal Investigator: Zhaojun Mo, Master

IPDSharing

Plan to Share IPD:

References

Citations:

Links:

Available IPD/Information:
